# Photodynamic Therapy for Atherosclerosis

**DOI:** 10.3390/ijms25041958

**Published:** 2024-02-06

**Authors:** Wiktoria Mytych, Dorota Bartusik-Aebisher, Aleksandra Łoś, Klaudia Dynarowicz, Angelika Myśliwiec, David Aebisher

**Affiliations:** 1 Students English Division Science Club, Medical College of the University of Rzeszów, 35-959 Rzeszów, Poland; wiktoriamytych@gmail.com (W.M.); aleksandralos1999@gmail.com (A.Ł.); 2 Department of Biochemistry and General Chemistry, Medical College of the University of Rzeszów, 35-959 Rzeszów, Poland; dbartusikaebisher@ur.edu.pl; 3 Center for Innovative Research in Medical and Natural Sciences, Medical College of the University of Rzeszów, 35-310 Rzeszów, Poland; kdynarowicz@ur.edu.pl (K.D.); amysliwiec@ur.edu.pl (A.M.); 4 Department of Photomedicine and Physical Chemistry, Medical College of the University of Rzeszów, 35-959 Rzeszów, Poland

**Keywords:** atherosclerosis, photodynamic therapy, plaques

## Abstract

Atherosclerosis, which currently contributes to 31% of deaths globally, is of critical cardiovascular concern. Current diagnostic tools and biomarkers are limited, emphasizing the need for early detection. Lifestyle modifications and medications form the basis of treatment, and emerging therapies such as photodynamic therapy are being developed. Photodynamic therapy involves a photosensitizer selectively targeting components of atherosclerotic plaques. When activated by specific light wavelengths, it induces localized oxidative stress aiming to stabilize plaques and reduce inflammation. The key advantage lies in its selective targeting, sparing healthy tissues. While preclinical studies are encouraging, ongoing research and clinical trials are crucial for optimizing protocols and ensuring long-term safety and efficacy. The potential combination with other therapies makes photodynamic therapy a versatile and promising avenue for addressing atherosclerosis and associated cardiovascular disease. The investigations underscore the possibility of utilizing photodynamic therapy as a valuable treatment choice for atherosclerosis. As advancements in research continue, photodynamic therapy might become more seamlessly incorporated into clinical approaches for managing atherosclerosis, providing a blend of efficacy and limited invasiveness.

## 1. Introduction

In 2016, the World Health Organization (WHO) reported that coronary vascular disease accounts for 31% of all deaths worldwide, representing 17.9 million deaths. The main factor in cardiovascular disease is atherosclerosis whose incidence is trending upwards. Therefore, atherosclerosis is a major life-threatening cardiovascular condition causing death from myocardial infarction and stroke [1,2]. Generally, the mechanisms of lipid metabolism contribute to atherosclerosis. The first study of atherosclerotic plaque took place in 1908 by Ignatowski, who studied plaques in the aortic walls of rabbits. Prior to this, in the early 19th century, Rudolph Virv described arterial inflammation [3,4,5]. Atherosclerosis is defined as a progressive inflammatory disease characterized by the accumulation of lipids in the walls of arterial vessels, a disease in which atherosclerotic plaques are deposited in the inner walls of the arteries. Atherosclerotic plaques lead to progressive lipid deposition, subsequent accumulation of T cells, macrophages in the arterial endothelium, and narrowing of the vessel lumen [6,7,8,9,10,11]. Current therapeutic strategies aim to modify risk factors, control inflammation, and stabilize vulnerable plaques. Statins, antiplatelet agents, and lifestyle interventions constitute the cornerstones of atherosclerosis management. Emerging research focuses on innovative approaches, including targeted drug delivery and immunomodulation, offering glimpses of a future where personalized and precise interventions may redefine the atherosclerosis treatment [12,13].

The vascular endothelial dysfunction of arteries is a serious factor in atherosclerotic symptoms. Atherosclerotic plaques containing apolipoprotein B-induced lipoprotein deposition from plasma can break off and cause thrombi. Apolipoprotein B triggers inflammatory processes. Some cytokines can cause atherosclerotic plaques to rupture and lead to the complete closure of the vessel lumen. Atherosclerotic plaques usually form asymptomatically at a young age and progress with age, giving a variety of symptoms. The early detection of atherosclerotic plaques is a priority [14,15]. Currently, biomarkers are used to verify atherosclerotic lesions. Most of them are limited to the myocardium only. The main biomarkers under consideration are myoglobin, troponin T, troponin I, and the muscle fraction of creatine kinase. Researchers also emphasize the importance and significant influence of myeloperoxidase, pregnancy plasma protein A, albumin, lipoprotein-associated phospholipase A2, interleukin 18, and CD40 receptor ligand [16,17,18]. Imaging diagnostics, primarily angiography and Doppler ultrasound, are also used. Many genetic and environmental factors are considered to be causes of atherosclerosis [19]. The primary risk factors are hypertension, diabetes, obesity, smoking, hypercholesterolemia, physical inactivity, chronic stress, age, and unhealthy diets. Scientists agree that all risk factors contribute to the pathogenesis of atherosclerotic lesions [20,21,22,23,24,25].

### 1.1. Pathogenesis of Atherosclerosis

**Figure 1:**
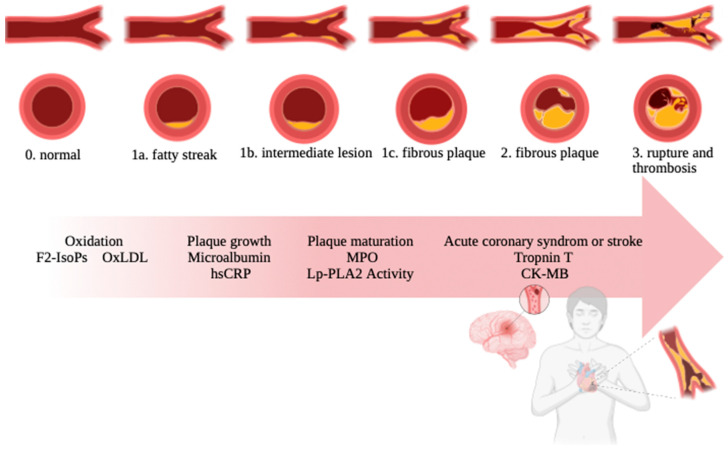
Arteriosclerosis process. The individual stages of atherosclerosis are fatty streak, intermediate lesion, fibrous plaque, rupture, and thrombosis.

Atherosclerosis plaque formation (Figure 1) emerges as a complex and dynamic process, weaving together molecular, cellular, and systemic influences. Understanding the intricacies of plaque development is paramount in the pursuit of effective prevention and intervention strategies [26]. Atherosclerosis initiates with endothelial dysfunction, a pivotal event often triggered by risk factors such as hyperlipidemia, hypertension, and smoking. The disruption of the endothelial layer leads to the exposure of the underlying vascular smooth muscle cells circulating blood constituents, marking the commencement of plaque formation [27]. Reduced bioavailability of nitric oxide (NO), a vasodilator, and increased expression of adhesion molecules like vascular cell adhesion molecule-1 (VCAM-1) and intercellular adhesion molecule-1 (ICAM-1) create a proinflammatory and proatherogenic endothelial environment [28]. Central to atherosclerosis is the accumulation of low-density lipoprotein (LDL) within the subendothelial space [29]. Oxidized LDL becomes a beacon for monocytes, which traverse the endothelium, attracting them to the site of injury. Hypercholesterolemia is one of the disorders that stimulate the production of monocytes from the bone marrow [30]. Monocytes arise from myeloid progenitor cells in the bone gist [31]. The accumulation of LDL in the bloodstream leads to its infiltration across the endothelium into the intima, primarily facilitated by transcytosis involving receptors SR-B1 and ALK1. This process occurs in conjunction with caveolae, emphasizing the significance of caveolae-dependent LDL uptake in LDL transcytosis [32]. Trapped LDL particles undergo oxidation in the subendothelial space, promoting atherosclerotic plaque development. Inflammation, oxidative stress, and enzymatic activity contribute to LDL oxidation, resulting in minimally modified (mmLDL) or extensively oxidized (oxLDL) forms with distinct biological activities. Extensively oxidized LDLs, unrecognizable by LDL receptors, contribute to inflammation and atherosclerosis through scavenger receptor uptake [33,34,35,36].

**Figure 2:**
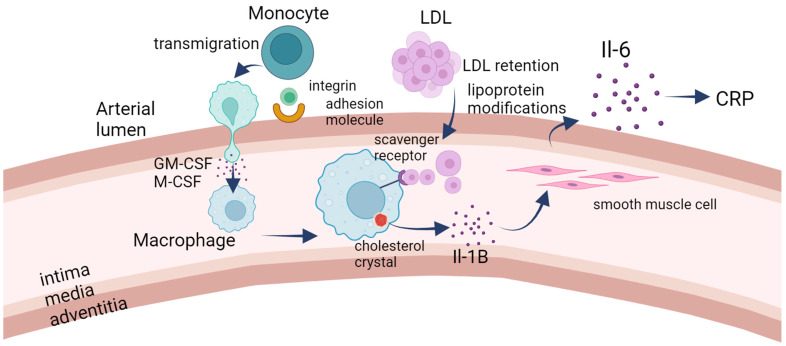
The immunology side of atherosclerosis. LDL retention serves as the initial trigger for the development of atherosclerosis. The subendothelial accumulation of lipoproteins prompts the upregulation of adhesion molecules on the endothelial surface, facilitating the recruitment of monocytes to the forming lesion. Subsequently, monocytes transmigrate into the subendothelial space, where they differentiate into macrophages in response to specific signals. Notably, smooth muscle cells have the capacity to undergo transdifferentiation into macrophage-like cells within the developing lesion. The scavenger–receptor-mediated uptake of lipoproteins by macrophages leads to the formation of foam cells, characterized by the intracellular accumulation of lipids. Cholesterol crystals may form within these foam cells, triggering the release of IL-1B. IL-1B, in turn, stimulates smooth muscle cells to produce IL-6. Both IL-1B and IL-6 exert proinflammatory effects, contributing to the inflammatory milieu within the atherosclerotic lesion. Additionally, circulating IL-6 may signal to the liver, prompting the production of C-reactive protein (CRP), which serves as a marker of inflammation. In summary, the cascade of events initiated by LDL retention involves endothelial activation, monocyte recruitment and differentiation, foam cell formation, and the release of proinflammatory cytokines. This inflammatory environment plays a crucial role in the progression of atherosclerosis, a condition characterized by the buildup of plaque within arterial walls.

Endothelial activation, triggered by proinflammatory stimuli, leads to phenotypic modulation, termed type II activation. This sustained activation induces a complex inflammatory response involving increased NF-kB production, upregulation of adhesion molecules, chemokines, and prothrombotic mediators, fostering monocyte recruitment into the intima [37]. Upon infiltration, monocytes differentiate into macrophages and engulf oxidized LDL, forming foam cells rich in cholesterol esters. The accumulation of cholesterol crystals activates the NLRP3 inflammasome, intensifying inflammation [38,39,40,41]. Macrophages can stimulate or suppress inflammation. This mechanism is essential in the functioning of the body, especially in fighting infections. The engorged macrophages, known as foam cells, constitute a hallmark of early atherosclerotic lesions. Foam cells release proinflammatory cytokines, including tumor necrosis factor-alpha (TNF-α) and interleukin-1 (IL-1) [42]. These cytokines propagate inflammation, attracting additional immune cells and perpetuating the atherogenic process. This cascade of events recruits additional immune cells (Figure 2), further promoting inflammation within the arterial wall [43,44,45].

The inflammatory milieu contributes to the progression of atherosclerosis by fostering oxidative stress and perpetuating endothelial dysfunction. Activated macrophages release growth factors such as platelet-derived growth factor (PDGF), stimulating the migration of vascular smooth muscle cells (VSMCs) from the media to the intima [46]. VSMCs undergo proliferation, contributing to the formation of a fibrous cap overlying the atherosclerotic plaque. The cap, composed of VSMCs and Extracellular Matrix (ECM), acts as a subendothelial barrier, preventing the exposure of the necrotic core to circulating coagulation factors. Persistent mitogen production hinders VSMC transition back to the contractile phenotype, facilitating lesion development. Oxidative stress is a hallmark of atherosclerosis, fueled by the activation of NADPH oxidase and the oxidative modification of LDL [47,48]. This results in the generation of ROS, including superoxide radicals and hydrogen peroxide, contributing to endothelial dysfunction, lipid peroxidation, and amplification of the inflammatory response. The fibrous cap’s characteristics determine plaque stability [49,50,51]. Plaque vulnerability arises when the delicate equilibrium between inflammation, cell proliferation, and matrix deposition is disrupted. As the plaque matures, it undergoes structural alterations. The fibrous cap may become thin and vulnerable, increasing the risk of rupture. Continued infiltration of lipids, immune cells, and sustained inflammatory signaling contribute to the formation of advanced atherosclerotic lesions [52,53]. Atheroma plaques form in areas of low Wall Shear Stress, causing endothelial dysfunction and eccentric plaque growth. Outward vessel remodeling initially occurs but perpetuates low-WSS conditions, making plaques rupture-prone [54]. Vulnerable plaques have a large necrotic core, a thin fibrous cap, and increased inflammation due to continuous exposure to a pro-atherogenic environment. The fibrous cap’s integrity, influenced by VSMC death and inflammation, determines plaque vulnerability [55].

The rupture of a vulnerable plaque exposes its thrombogenic core, triggering platelet activation and aggregation. Thrombus formation within coronary or cerebral vessels can result in acute clinical events such as myocardial infarction or stroke, marking critical points in the progression of atherosclerosis [56,57]. Efforts to resolve the plaque involve macrophage phagocytosis of cellular debris. However, chronic inflammation may lead to unresolved plaque components and calcium deposition, contributing to plaque stability but compromising its flexibility [58,59]. It is also worth noting that atherosclerosis susceptibility is influenced by genetic factors, with polymorphisms in genes related to lipid metabolism (e.g., APOE), inflammation (e.g., IL-6), and vascular function (e.g., eNOS) playing significant roles. Epigenetic modifications, including DNA methylation and histone acetylation, further regulate the expression of genes associated with atherosclerosis [60,61,62].

### 1.2. Biomarkers in Atherosclerosis

**Figure 3:**
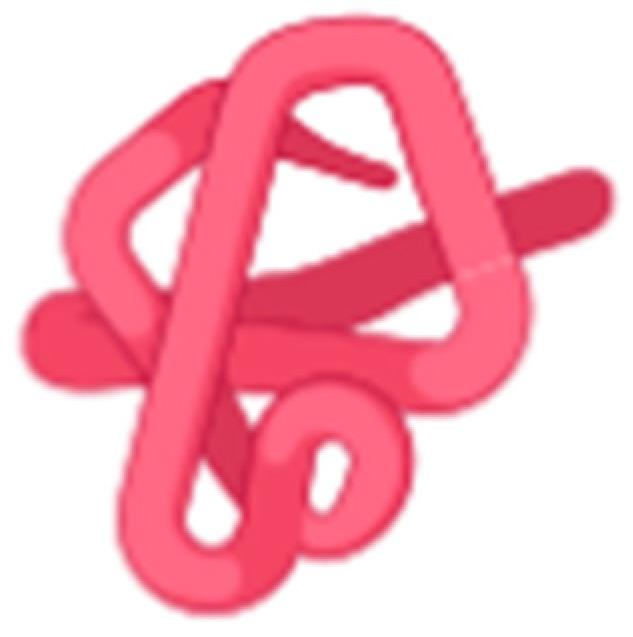
Myoglobin.

Myoglobin (Figure 3) is a protein found in muscle tissues that plays a crucial role in oxygen storage and transport within muscle cells. Myoglobin has been implicated in oxidative stress and inflammation. Oxidative stress occurs when there is an imbalance between the product of reactive oxygen species (ROS) and the body’s capability to neutralize them. Reactive oxygen species can contribute to the inflammation and damage of arterial walls, a key feature of atherosclerosis [63]. Additionally, myoglobin may be involved in the recruitment and activation of immune cells, such as macrophages, which play a role in the inflammatory response associated with atherosclerosis. The exact mechanisms by which myoglobin influences atherosclerosis are not fully understood, and research in this area is ongoing [64,65,66].

**Figure 4:**
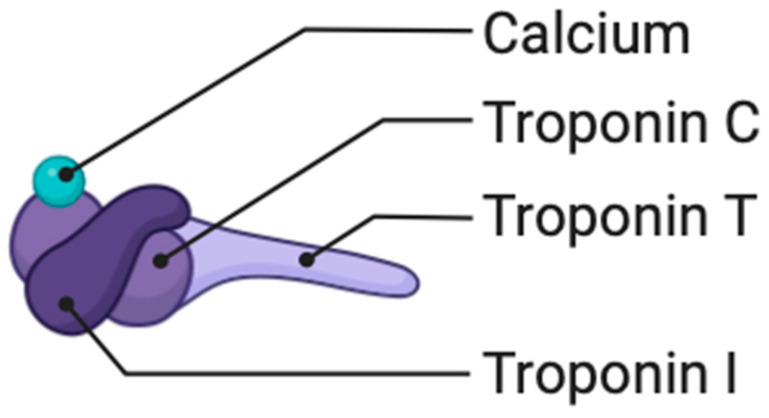
Tropnin complex.

Troponin T (Figure 4) is a protein primarily known for its role in muscle contraction, particularly in cardiac muscle. While troponin T is traditionally associated with cardiac muscle and is a well-established biomarker for diagnosing myocardial infarction, its direct involvement in atherosclerosis, a process of plaque buildup in arteries, is not as extensively studied [67]. However, there is some emerging research suggesting potential links between troponin T and cardiovascular diseases, including atherosclerosis. Troponin T may be implicated in the inflammatory and pathological processes that contribute to plaque formation in atherosclerosis. Inflammation plays a significant role in the development and progression of atherosclerosis, and there is ongoing research to understand the specific roles of various proteins, including troponin T, in these inflammatory pathways [68,69,70].

Troponin I (Figure 4) may be implicated in the inflammatory pathways associated with atherosclerosis, possibly contributing to the vascular damage that occurs during the development of plaques [71].

Creatine kinase (CK), also known as creatine phosphokinase (CPK), is an enzyme found in various tissues, including skeletal muscles, heart muscles, and the brain. Creatine kinase catalyzes the conversion of creatine and adenosine diphosphate (ADP) into phosphocreatine and adenosine triphosphate (ATP), playing a crucial role in energy metabolism. There is a particular interest in the muscle fraction of creatine kinase, known as CK-MB (creatine kinase-MB) [72]. Creatine kinase MB is often associated with cardiac muscle damage and is a well-established marker for diagnosing myocardial infarction. The elevated levels of CK-MB in the blood are indicative of damage to heart muscle cells. While CK-MB is primarily associated with cardiac muscle, atherosclerosis is a condition that involves the buildup of plaques in the arteries, and its direct involvement in atherosclerosis is not as prominent. The release of CK-MB into the bloodstream can occur when there is damage to the heart muscle, which may happen in the context of ischemia or infarction associated with advanced atherosclerosis [73,74,75,76].

Myeloperoxidase (MPO) is an enzyme released by white blood cells, particularly neutrophils, as part of the immune response. It plays a role in the body’s defense against infections by generating ROS and promoting the formation of hypochlorous acid [77]. While MPO is a crucial component of the immune system, it has also been associated with inflammatory processes in various diseases, including atherosclerosis. In the context of atherosclerosis, MPO is thought to contribute to the oxidative modification of low-density lipoprotein (LDL) cholesterol, a key step in the development of atherosclerotic plaques [78]. The oxidative modification of LDL makes it more likely to be taken up by immune cells, leading to the formation of foam cells and the initiation of the inflammatory response within arterial walls. The elevated levels of MPO have been detected in atherosclerotic lesions, and studies suggest that it may serve as a marker for increased cardiovascular risk. MPO is considered a potential biomarker for assessing inflammation and oxidative stress associated with atherosclerosis, and its measurement in blood samples is being explored in clinical research [79,80].

Pregnancy-associated plasma protein A (PAPP-A) is an enzyme that plays a role in the regulation of insulin-like growth factor (IGF) activity. While its primary association is with pregnancy, where it is involved in the growth and development of the placenta, PAPP-A has also been studied in the context of atherosclerosis [81]. In atherosclerosis, increased levels of PAPP-A have been observed, and research suggests that it may be involved in the destabilization of atherosclerotic plaques. Atherosclerosis involves the buildup of plaques in the arterial walls, and the stability of these plaques is a critical factor in determining the risk of complications such as heart attacks or strokes [82]. PAPP-A may contribute to plaque instability by promoting the degradation of the fibrous cap that covers atherosclerotic lesions. A weakened fibrous cap is more prone to rupture, leading to the release of plaque contents into the bloodstream, which can trigger blood clot formation and result in cardiovascular events. As a result, PAPP-A is considered a potential biomarker for assessing the vulnerability of atherosclerotic plaques and predicting the risk of cardiovascular events. The elevated levels of PAPP-A in blood samples may be indicative of increased plaque instability and higher cardiovascular risk [83,84,85].

**Figure 5:**
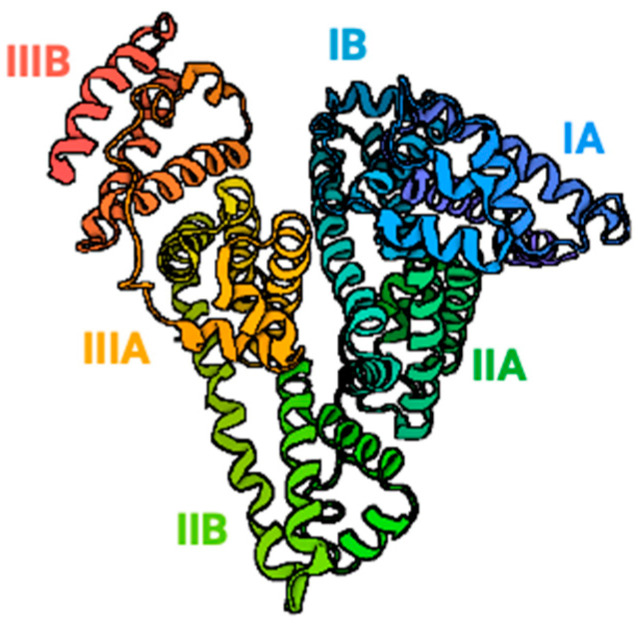
Albumin tertiary structure.

Albumin (Figure 5) is a protein commonly found in blood plasma and serves various physiological functions, including maintaining osmotic pressure and transporting substances such as hormones, fatty acids, and drugs [86]. While albumin itself is not directly implicated in the development of atherosclerosis, its levels and the presence of certain modifications to albumin can be relevant in assessing cardiovascular health. Researchers have explored associations between altered albumin levels and atherosclerotic risk factors [87]. For example, low serum albumin levels have been observed in individuals with cardiovascular disease, and hypoalbuminemia is considered a potential marker of systemic inflammation and malnutrition, which are factors that may contribute to the progression of atherosclerosis. Additionally, the oxidative modifications of albumin, such as carbonylation, have been implicated in the inflammatory processes associated with atherosclerosis. Oxidative stress plays a role in the initiation and progression of atherosclerotic plaques, and modified proteins, including albumin, may contribute to the overall inflammatory milieu [88,89,90].

Lipoprotein-associated phospholipase A2 (Lp-PLA2) is an enzyme that is associated with lipoproteins, particularly low-density lipoprotein (LDL) cholesterol. It plays a role in the inflammatory processes within arterial walls and has been studied in the context of atherosclerosis [91]. In atherosclerosis, Lp-PLA2 is thought to be involved in the modification of LDL particles, making them more prone to inflammation and contributing to the development of atherosclerotic plaques. The enzyme catalyzes the hydrolysis of oxidized phospholipids in LDL, releasing proinflammatory substances [92]. The elevated levels of Lp-PLA2 have been associated with increased cardiovascular risks. It is considered a potential biomarker for assessing the vulnerability of atherosclerotic plaques, as well as a marker of systemic inflammation. The measurements of Lp-PLA2 in blood samples are being explored in clinical research as a tool for predicting the risk of cardiovascular events, such as heart attacks or strokes [93,94].

**Figure 6:**
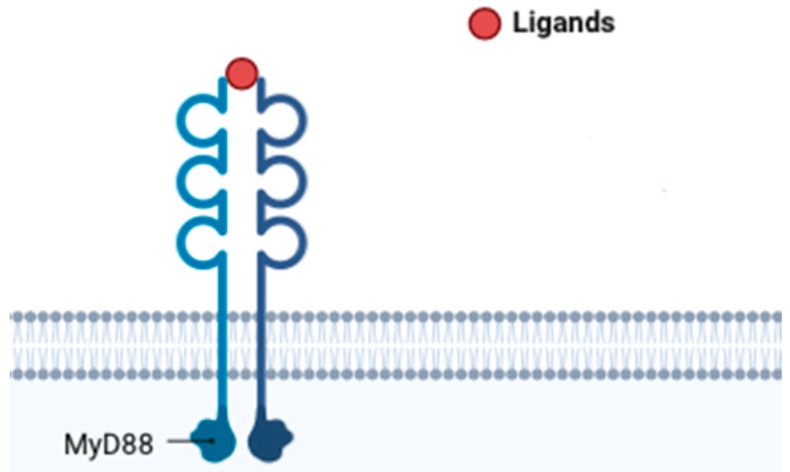
IL-18.

Interleukin-18 (IL-18) (Figure 6) is a proinflammatory cytokine involved in the vulnerable response. In the context of atherosclerosis, IL-18 has been implicated in the inflammatory processes that contribute to the development and progression of atherosclerotic plaques [95]. IL-18 is produced by various cells, including macrophages and endothelial cells, within the arterial walls. It can stimulate the release of other inflammatory molecules and contribute to the recruitment of immune cells, such as T lymphocytes, into atherosclerotic lesions [96]. This inflammatory response promotes the formation of atherosclerotic plaques, characterized by the buildup of cholesterol, immune cells, and other substances within the arterial walls. The elevated levels of IL-18 have been observed in individuals with atherosclerosis, and it is considered a potential biomarker for assessing the inflammatory status associated with cardiovascular disease [97]. Research suggests that IL-18 may play a role in plaque instability, making it a subject of interest in understanding the mechanisms underlying the complications of atherosclerosis, such as plaque rupture and thrombosis [98].

**Figure 7:**
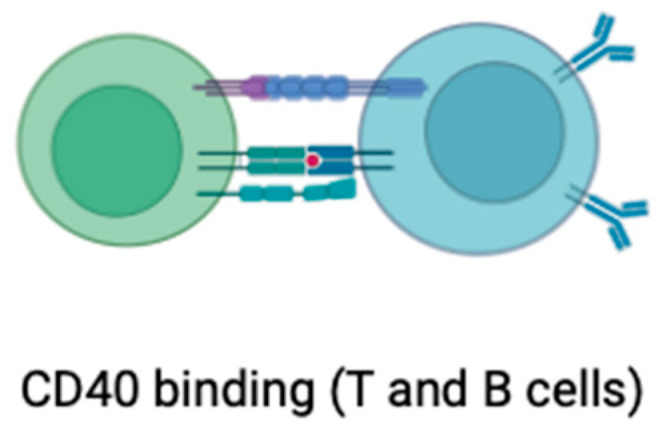
CD40.

The CD40 (Figure 7) receptor ligand, often referred to as CD40L or CD154, is a molecule expressed on the surface of activated immune cells, particularly T cells. Its interaction with the CD40 receptor on various cell types, including endothelial cells and immune cells, plays a critical role in immune responses and inflammation [99]. In the context of atherosclerosis, CD40L has been implicated in the inflammatory processes that contribute to the development and progression of atherosclerotic plaques. CD40L is involved in the activation of endothelial cells, which line the inner surface of blood vessels [100]. This activation leads to the increased expression of adhesion molecules and the recruitment of immune cells, such as monocytes and T cells, into the arterial walls. Within the atherosclerotic lesions, CD40L further stimulates the release of pro-inflammatory molecules, contributing to the formation of atherosclerotic plaques [101]. The elevated levels of CD40L have been observed in individuals with atherosclerosis, and research suggests that it may serve as a biomarker for assessing the inflammatory status associated with cardiovascular disease. Additionally, the CD40L/CD40 interaction is considered a potential target for therapeutic interventions aiming to modulate the inflammatory response in atherosclerosis [102,103,104,105].

## 2. Atherosclerosis Treatment and Photodynamic Therapy

Atherosclerosis treatment involves lifestyle changes, medications to control risk factors, and in some cases, interventional procedures or surgery. Lifestyle modifications include a heart-healthy diet, exercise, and smoking cessation. Medications such as statins, antiplatelet agents, and antihypertensives are commonly prescribed [106]. Interventional procedures like angioplasty and stenting can open narrowed arteries, while surgeries like bypass surgery or endarterectomy may be considered in severe cases. Emerging therapies include immunotherapy, gene therapy, and innovative approaches like photodynamic therapy. Treatment is tailored based on individual factors, emphasizing the importance of regular monitoring and adherence to minimize complications [107].

**Figure 8:**
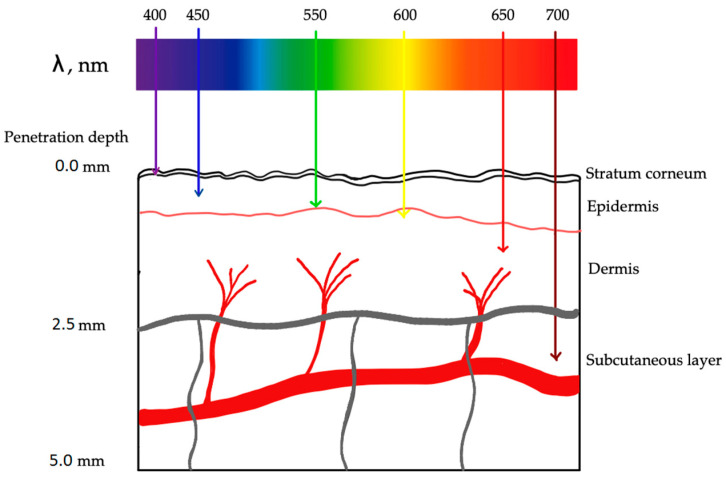
Photodynamic therapy in atherosclerosis. Each wavelength of light has a different depth of tissue penetration.

Cardiovascular diseases, particularly atherosclerosis, stand as formidable challenges to global health. In the ongoing quest for effective interventions, photodynamic therapy (PDT) has emerged as a beacon of hope, leveraging light-sensitive compounds to selectively target and stabilize atherosclerotic plaques [108]. The journey commences with the strategic administration of a photosensitizer, a molecular envoy designed with precision to selectively accumulate within atherosclerotic plaques. The photosensitizer’s choice hinges on its ability to distinguish between diseased and healthy tissues, marking the first step in PDT’s tailored approach. The photosensitizer’s trajectory is guided by the distinct characteristics of atherosclerotic plaques, such as increased vascular permeability [109]. Exploiting these unique features, the photosensitizer hones in on the target site, ensuring a concentrated payload within the very heart of the pathology. A crucial inflection point arises when the accumulated photosensitizer encounters light of a specific wavelength (Figure 8).

**Figure 9:**
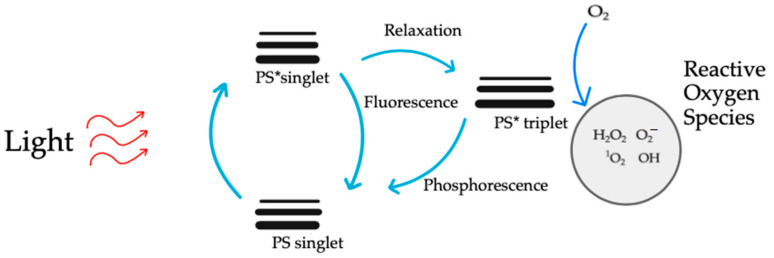
PDT mechanism. The entire process of destroying cancer cells during PDT may occur according to two reactions. Both mechanisms can occur simultaneously. The PSs become active (PS*) when exposed to a certain wavelength of light, transferring their energy to molecules of oxygen, which then change oxygen from its initial state into a lethal singlet state, producing singlet oxygen (^1^O_2_) that kills tumor cells. As a result, ROS are crucial to PDT’s ability to eliminate malignancies. ^1^O_2_ is formed by the excitation of the triplet oxygen molecule. It belongs to one of the ROS. ROS are products of successive stages of reduction in the oxygen molecule. Oxygen reduction and excitation products are more reactive than triplet oxygen. ^1^O_2_ is the major cytotoxic agent involved in PDT. The mechanism of action of ^1^O_2_ is based on its high chemical reactivity. The excited ^1^O_2_ molecule aims to reduce the energy state, which is achieved in the process of oxidation of various substances. It reacts readily with lipids, proteins, and nucleic acids, changing their structure.

The activation of the photosensitizer by light propels it into an excited state, setting the stage for the cascade of events that define PDT’s therapeutic prowess [110]. In this excited state, the photosensitizer transforms into a molecular alchemist, engaging in a dance with molecular oxygen (Figure 9). This intricate interplay results in the generation of reactive oxygen species (ROS), notably singlet oxygen [111]. These ROS are the molecular architects of PDT, orchestrating the targeted cellular destruction that defines its mechanism. The unleashed ROS embark on a mission of selective cellular devastation within the atherosclerotic plaque. Endothelial cells and smooth muscle cells, which are integral components of the plaque architecture, bear the brunt of this orchestrated assault [112]. The high reactivity of ROS induces oxidative stress, precipitating a cascade of events that culminate in cellular ablation. As ROS emerge, they usher in a state of oxidative stress within the targeted cells and tissues [113].

**Figure 10:**
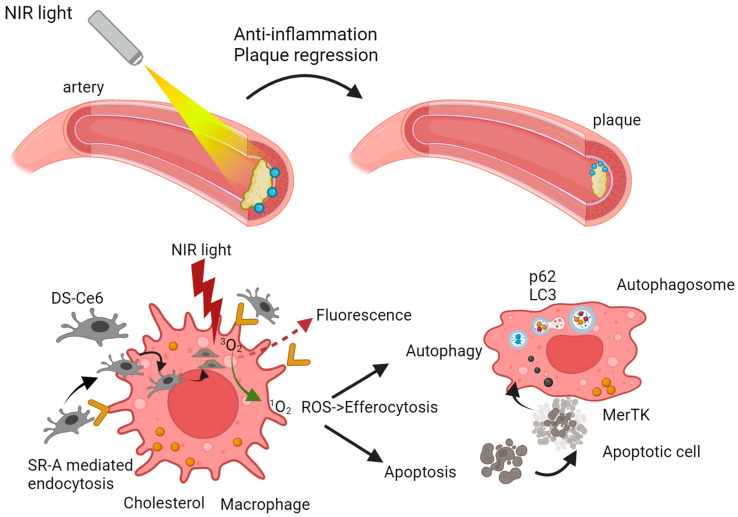
Photoactivation within an atherosclerotic artery.

This oxidative stress, characterized by an imbalance between ROS production and cellular defense mechanisms, becomes the driving force behind PDT’s therapeutic effects. One of the key movements in this process is lipid peroxidation. Singlet oxygen initiates a chain reaction leading to the oxidative degradation of lipids in cell membranes [114]. The resulting cacophony of reactive aldehyde species, notably malondialdehyde, contributes to cellular damage and sets the tone for further cellular responses. As the process progresses, ROS extend their influence on the intricate world of proteins. Oxidative stress can modify proteins, altering their structure and function. The formation of carbonyl groups in proteins becomes equal to functional impairment and cellular dysfunction [115]. The process reaches a crescendo with the profound impact of ROS on DNA. Single-strand and double-strand breaks in the DNA sequence become a poignant movement, activating cellular repair mechanisms. However, excessive damage may lead to overwhelming responses, tipping the balance toward programmed cell death [116]. Mitochondrial proximity to ROS generation sites renders them susceptible to oxidative stress. Reactive oxygen species-induced damage to mitochondrial components becomes a pivotal movement, impacting energy production and triggering apoptotic pathways [117]. This cellular stage responds to ROS-induced oxidative stress in multifaceted ways. Low levels of oxidative stress may activate survival pathways and cellular repair mechanisms, embodying resilience. However, the culmination of oxidative stress can lead to irreversible damage, propelling the cellular changes toward programmed cell death [118]. Beyond its direct cellular impact, PDT introduces an intriguing dimension with its immunomodulatory effects. The ROS-induced cellular damage triggers an inflammatory response, a controlled conflagration within the treated area. This interplay between inflammation and healing mechanisms introduces complexity to the therapeutic landscape, offering potential avenues for immune-mediated plaque resolution [119]. As the ablated cells undergo programmed cell death (Figure 10), macrophages and other phagocytic cells enter, engaging in the clearance of cellular debris. This orchestrated removal process contributes to the resolution of the atherosclerotic plaque [120]. Simultaneously, the potential for tissue healing and restructuring unfolds as the body’s regenerative mechanisms come into play. PDT may exert anti-proliferative effects on vascular smooth muscle cells, inhibiting their excessive proliferation and migration. This property can contribute to stabilizing the plaque and preventing restenosis [121,122].

The mechanism of PDT in atherosclerosis unfolds as a symphony of precision and selectivity. From the strategic deployment of photosensitizers to the choreography of ROS-induced cellular ablation and controlled inflammation, PDT navigates the complexities of atherosclerosis with finesse. As research progresses, the unraveling of these intricate processes promises to illuminate the path forward in the quest for effective and targeted atherosclerosis treatment [123]. The key advantage of PDT lies in its ability to selectively target atherosclerotic lesions while sparing healthy tissues. This makes it a promising strategy for plaque-specific intervention. While preclinical studies have demonstrated encouraging results, ongoing research and clinical trials are necessary to further refine the technique, optimize treatment protocols, and evaluate long-term safety and efficacy [124]. The potential of PDT in combination with other therapeutic modalities adds to its versatility, offering a novel avenue for addressing atherosclerosis and its associated cardiovascular risks. PDT not only stands as a treatment modality but also as a beacon of hope, guiding us toward a future where cardiovascular health is illuminated by the light of innovative therapeutic strategies. While the mechanism of PDT in atherosclerosis paints an optimistic picture, challenges persist on the road to clinical translation [125]. Optimizing the properties of photosensitizers for enhanced plaque specificity, refining light delivery systems, and navigating the delicate balance between controlled inflammation and collateral damage are ongoing priorities for researchers.

Statin therapy, a cornerstone in cardiovascular care, focuses on reducing blood cholesterol levels, particularly low-density lipoprotein cholesterol (LDL-C). By inhibiting HMG-CoA reductase, the enzyme responsible for cholesterol synthesis, statins not only lower LDL-C but also exert anti-inflammatory and antioxidant effects [126,127]. These properties make statins valuable in stabilizing atherosclerotic plaques and preventing cardiovascular events. Complementing statin therapy, photodynamic therapy introduces a targeted and selective dimension to atherosclerosis treatment [128]. The synergy between statin therapy and PDT is evident in their complementary mechanisms of action. Statins address the systemic factors contributing to atherosclerosis, reducing cholesterol levels and stabilizing plaques [129]. Meanwhile, PDT provides a localized intervention, directly targeting the atherosclerotic lesion and promoting plaque regression [130]. Moreover, the anti-inflammatory effects of statins may synergize with PDT, potentially enhancing the therapeutic response. The dual approach addresses not only the lipid-related aspects of atherosclerosis but also the cellular and inflammatory components, offering a more comprehensive solution to the multifaceted nature of the disease [131]. Integrating PDT with statin therapy presents a holistic and nuanced approach to atherosclerosis management. By combining the systemic benefits of statins with the targeted precision of PDT, this comprehensive strategy aims to address the diverse facets of atherosclerosis, paving the way for enhanced patient outcomes and improved cardiovascular health. As research advances, this integrative approach may herald a new era in the treatment of cardiovascular diseases, offering a beacon of hope for those grappling with the complexities of atherosclerosis. Despite the promise of combining PDT and statin therapy, challenges persist. Optimizing the choice of photosensitizers for enhanced plaque specificity, refining light delivery systems, and conducting thorough safety assessments are ongoing priorities. Additionally, understanding the long-term effects of combined therapy and its impact on cardiovascular outcomes requires further investigation.

### Nanoparticles 

Nanoparticles are minuscule particles with dimensions typically ranging from 1 to 100 nanometers and have emerged as promising tools in the field of medicine, offering unique opportunities for targeted drug delivery and diagnostic applications [132]. In the context of atherosclerosis, nanoparticles have garnered attention for their potential to address key challenges in treatment. Atherosclerosis poses a significant global health burden, contributing to conditions such as heart attacks and strokes [133]. Traditional therapeutic approaches often face limitations, including systemic side effects and insufficient targeting of affected areas. Nanoparticles, due to their size and tunable properties, present an innovative avenue for enhancing the precision and efficacy of treatments for atherosclerosis [134]. The role of nanoparticles in combating atherosclerosis includes examining their ability to navigate through the circulatory system, selectively targeting atherosclerotic lesions, and delivering therapeutic payloads with improved efficiency [135]. By harnessing the unique properties of nanoparticles, researchers aim to revolutionize the treatment landscape of atherosclerosis, offering new hope for more effective and targeted interventions in the battle against cardiovascular disease [136]. Table 1 shows common nanoparticles in atherosclerosis.

## 3. A Review of the Literature

### 3.1. In Vitro

An investigation into optimal conditions for PDT using Upconversion Nanoparticles (UCNPs)-Ce6 yielded significant insights into treating atherosclerosis. The research identified a specific dosage of UCNPs-Ce6, combined with precise laser parameters, as effective in reducing lipid storage in THP-1 macrophage-derived foam cells. This reduction was linked to the promotion of cholesterol efflux, with the study revealing autophagy induction as a mechanism mediated by ABCA1 and involving the suppression of the PI3K/Akt/mTOR pathway [145]. The application extended to peritoneal macrophage-derived foam cells, emphasizing the potential of UCNPs-Ce6-mediated PDT in atherosclerosis treatment.

In a novel approach, bTiO2-HA-p nanoprobes demonstrated synergistic effects on lipid metabolism within atherosclerotic foam cells. These nanoprobes exhibited promising photothermal and photodynamic properties, along with excellent biocompatibility, offering enhanced anti-atherosclerosis therapeutic effects without inducing excessive cell apoptosis or necrosis [146].

Spyropoulos-Antonakakis et al. [147] introduced G0 PAMAM dendrimers and G0 PAMAM/ZnPc conjugates to carotid tissues, revealing significant modifications in nanoscale texture characteristics. The study emphasized the need for future investigations on various factors influencing dendrimer penetration across the endothelial barrier [147].

Macrophage-targeted PDT using chlorin(e6) (ce6) and maleylated bovine serum albumin (BSA-mal) conjugates demonstrated selective targeting of macrophages. The study highlighted differences in macrophage activation and scavenger receptor class A (SRA) expression between murine macrophage tumor cell lines, indicating the influence of macrophage activation status on PDT efficacy [148].

A comprehensive examination of curcumin-mediated photodynamic therapy (CUR-PDT) on Vascular Smooth Muscle Cells (VSMCs) revealed optimal treatment conditions and identified autophagy as a key mechanism underlying therapeutic effects, suggesting CUR-PDT’s potential for treating atherosclerosis [149].

UCNPs-Ce6-mediated PDT targeting mitochondria showed promising outcomes in reducing macrophage infiltration in atherosclerotic plaques, indicating its potential as a treatment for atherosclerosis [150].

The use of a novel photosensitizer, ATMPyP, in combination with PDT demonstrated selective accumulation in atherosclerotic plaques, enhancing therapeutic effects [151].

The intravascular delivery of photosensitizers using oxidatively modified low-density lipoprotein (OxLDL) as a carrier showcased improved selectivity for atherosclerotic lesions, emphasizing the potential of targeted delivery systems in optimizing PDT outcomes [152].

A study by Spyropoulos-Antonakakis et al. focused on a dextran-bovine serum albumin conjugate (DB) used to create a nanoemulsion loaded with UCNPs and Ce6 (UCNPs-Ce6@DB). This approach facilitated recognition and binding to scavenger receptor class A (SR-A) on macrophages, promoting uptake by macrophage-derived foam cells in atherosclerotic lesions. UCNPs-Ce6@DB-mediated PDT enhanced ROS generation, induced autophagy, upregulated ABCA1 expression for cholesterol efflux, and suppressed pro-inflammatory cytokine secretion, showing promise in inhibiting plaque formation in AS mouse models [153].

The impact of pyropheophorbide-α methyl ester (MPPa)-mediated PDT on apoptosis and inflammation in murine macrophage RAW264.7 cells demonstrated the potential to induce apoptosis and alleviate inflammation, presenting a promising therapeutic approach for atherosclerosis [154].

The capability of Photodynamic Diagnosis (PDD) and PDT using chlorin e6 for detecting atherosclerotic plaque was explored in a study with human aorta and coronary artery specimens. Chlorin e6 accumulation in atheromatous plaque suggested specificity for discriminating between atheromatous and normal/calcified segments, providing a potential tool for real-time imaging and targeted therapy in various forms and stages of atherosclerosis [155].

### 3.2. In Vivo in Mice Models

In a comprehensive exploration of advanced therapeutic approaches for atherosclerosis, researchers have embarked on multiple studies with a focus on precision and targeted intervention. One notable investigation centered on stable iron-based nanoparticles (FeCNPs) modified with Ce6 and 3,4-DA for targeted atherosclerosis treatment [156]. These FeCNPs demonstrated effective plaque accumulation, showcasing significant reductions at both low and high doses, with superior outcomes observed at lower doses. Biodistribution tracking confirmed sustained FeCNP retention in plaques, accompanied by positive changes in plaque composition, including improvements in lipid-rich areas and reduced necrotic cores. Moreover, the FeCNPs exhibited a targeted approach towards MCP1, resulting in the reduction in macrophages and key markers associated with atherosclerosis progression. The study’s nuanced findings suggested potential avenues for improved targeting through molecule modifications, emphasizing the promising therapeutic potential of FeCNPs in atherosclerosis treatment. Additionally, the investigation explored the efficacy of chemiexcited Photodynamic Therapy (PDT) using Fe^3+^–catechol cross-linked CPPO-loaded polymeric nanoparticles, presenting promising prospects for treating atherosclerosis and underscoring the importance of refining plaque-targeting ability for optimal therapeutic effectiveness [156].

In a distinct approach, researchers developed theranostic nanoparticles targeting atherosclerotic plaques using osteopontin (OPN). By encapsulating a photosensitizer (IR780) and a chemo-drug (TPZ), these OPN-targeted nanoparticles selectively accumulated in foamy macrophages within vulnerable plaques. Photodynamic therapy (PDT) using these nanoparticles demonstrated reduced plaque area, increased stability, and improved blood perfusion, indicating a promising approach for precise imaging and effective treatment of atherosclerosis [157].

Han X. et al. [158] delved into the impact of upconversion nanoparticles encapsulating chlorin e6 (UCNPs-Ce6)-mediated PDT on M1 peritoneal macrophages. The research demonstrated that ROS generated by UCNPs-Ce6-mediated PDT induced autophagy, inhibiting the expression of pro-inflammatory factors in these macrophages. The study established the activation of autophagy through the PI3K/AKT/mTOR signaling pathway, leading to a reduction in inflammatory cytokines. The findings suggest a potential therapeutic avenue for mitigating inflammation associated with atherosclerosis [158].

In a nuanced exploration of photosensitizer distribution in atherosclerotic plaques, a study revealed variations based on lesion characteristics. Plaques in ApoE−/− mice exhibited high lipid content, necrotic cores, and endothelial lining. PDT resulted in reduced vasoconstriction and relaxation, indicating an impact on arterial function [159].

A study involving the photosensitizer mTHPC loaded into Ben-PCL-mPEG micelles addressed the challenge of premature release from micelles, focusing on improving stability for enhanced macrophage selectivity. The successful achievement of this goal, coupled with the effective targeting of atherosclerotic plaques, suggested the potential of mTHPC-loaded micelles for selective macrophage photocytotoxicity [160].

### 3.3. In Vivo on Rabbits

Waksman R. et al. [161] investigated the impact of PDT on regional atherosclerosis using cholesterol-fed rabbits. Their study compared PhotoPoint PDT treatment, photosensitizer alone, and light alone, revealing a substantial reduction in plaque progression by 35% at 7 days and 53% at 28 days. Importantly, PDT demonstrated the ability to decrease macrophages by 98% at 7 days and sustained a reduction by 92% at 28 days, without causing adverse vascular effects. This research highlighted PDT’s potential for treating acute coronary syndromes by reducing inflammation and promoting plaque stabilization [161].

Another study focused on the accumulation of the photosensitizer hematoporphyrin derivative (HPD) in atherosclerosis. The results demonstrated uniform HPD accumulation in injured media, suggesting PDT as a promising avenue for inhibiting smooth muscle cell growth in atherosclerotic lesions and potentially preventing restenosis following angioplasty [162].

Further exploration into various photosensitizers such as CASPc [163], NPe6 [164], and Lu-Tex [165] showcased their selective accumulation in atheromatous plaques. These findings underscored the versatility of PDT in utilizing different photosensitizers for the targeted treatment of atherosclerosis.

An investigation into texaphyrins, specifically Lutetium texaphyrin (PCI-0123), demonstrated selective localization in both cancer and atheromatous plaques. The ability of texaphyrins to selectively target diseased tissues suggested their potential for inducing tissue-specific damage, opening avenues for further exploration in the treatment of atherosclerosis [166].

In a recent study, an innovative approach was demonstrated for enhanced targeting in atherosclerotic plaques using a scavenger–receptor-directed photosensitizer (PS) conjugate named MA-ce6 [167]. This approach showed increased accumulation of MA-ce6 in macrophage-rich plaques, indicating potential benefits for targeted PDT. Future research aims to correlate PS conjugate accumulation with macrophage localization and investigate the therapeutic benefits of PDT through the intravascular delivery of red light in living rabbits [168].

Another noteworthy study focused on enhancing PDT efficacy for cardiovascular diseases by delivering photosensitizers specifically to atherosclerotic lesions. Utilizing oxidatively modified low-density lipoprotein (OxLDL) as a targeted carrier for the photosensitizer aluminum phthalocyanine chloride (AlPc), the study demonstrated light-dependent cytotoxicity in macrophages incubated with OxLDL-AlPc. This targeted delivery system holds promise for improving the therapeutic benefits of PDT in cardiovascular diseases [168].

Exploring PDT for treating inflamed atherosclerotic plaque in a rabbit model, a study utilized 5-aminolevulinic acid (ALA) as a photosensitizer. Positive correlations between ALA-derived protoporphyrin IX (PpIX) fluorescence and macrophage content in the plaque were observed. Photodynamic therapy resulted in a significant reduction in the plaque area, macrophage content, and the depletion of smooth muscle cells, indicating the potential for inhibiting plaque progression [169].

The potential of PDT for preventing in-stent restenosis after angioplasty was investigated in normal rabbits. Photodynamic therapy before stent deployment led to almost complete medial cell ablation at 3 days and significantly inhibited in-stent restenosis at 28 days, suggesting PDT as a promising adjuvant therapy to percutaneous intervention in patients with vascular disease [170].

In the realm of pharmacokinetics, a study compared the pre-photosensitizers ALA and ALA-ethyl ester in a rabbit model with post-balloon injured arteries. ALA demonstrated a higher selective build-up in atheromatous plaque compared to ALA-ethyl, suggesting potential effectiveness for endovascular PDT of atheromatous plaque. However, further optimization is needed to prevent potential restenosis [171].

The importance of the treatment field in PDT for preventing intimal hyperplasia (IH) in the rat carotid artery was emphasized in a study. While PDT initially prevented IH by depleting medial smooth muscle cells, the study concluded that including the entire injured artery or a section of an uninjured margin in the treatment field is crucial for the effective prevention of IH with PDT [172].

Finally, Motexafin lutetium (Lu-Tex), a PDT agent that localizes in atheromatous plaque, demonstrated significant potential in treating accelerated atherosclerosis associated with transplantation in a rodent allograft model of graft coronary artery disease (GCAD). Lu-Tex-mediated PDT significantly reduced affected vessels and intimal proliferation compared to control groups, suggesting its efficacy in managing transplantation-related atherosclerosis [173].

### 3.4. In Vivo on Pigs

In a study conducted on a large animal model, the objective was to develop a percutaneous method for applying PDT to iliac and coronary arteries and assess its impact on arterial remodeling and the intimal hyperplastic response to balloon injury. The results indicated that PDT treatment exerted a favorable influence on the arterial response to balloon injury in both coronary and peripheral circulations. This outcome presents a promising approach to address restenosis following endovascular procedures, providing potential insights for enhancing the effectiveness of percutaneous interventions [174].

Furthermore, in an exploration of intravascular light delivery for arterial PDT, a study employed a balloon catheter in a pig non-injury model. The study demonstrated that PDT delivered via a standard percutaneous transluminal angioplasty (PTA) balloon effectively depleted the vascular smooth muscle cell population within the arterial wall without complications. This suggests the potential of intravascular PDT to reduce the incidence of restenosis post-angioplasty, showcasing its viability as a minimally invasive intervention for addressing arterial complications [175].

### 3.5. In Vivo on Humans

Photodynamic therapy has been explored as an adjuvant to femoral percutaneous transluminal angioplasty (PTA) in a clinical study involving seven patients. These individuals, who had previously undergone conventional angioplasty at the same site resulting in restenosis or occlusion, received PDT sensitized with oral 5-aminolaevulinic acid. Red light (635 nm) was delivered to the angioplasty point via a ray fiber within the angioplasty balloon. The study demonstrated that all patients tolerated the procedure well without adverse complications, maintained sustained asymptomatic status, and had patent vessels without restenosis during the study interval. These findings suggest that endovascular PDT might be a safe and potentially effective method to reduce restenosis following angioplasty. Further investigation through a randomized controlled trial is warranted to validate these promising results [176].

Motexafin lutetium, an investigational photosensitizer, underwent evaluation in a clinical trial involving 47 patients with arterial stenosis. The trial suggested the safety and potential benefits of motexafin lutetium therapy in the context of atherosclerosis, paving the way for further clinical exploration [177].

In another research endeavor, the applicability of Benzoporphyrin derivative (BPD) as a potent photosensitizer for treating atherosclerosis through PDT was investigated. The study delved into the uptake of BPD in atherosclerotic plaque, examining both in vitro and in vivo scenarios. Atherosclerotic human arteries and induced atherosclerotic miniswine were exposed to different concentrations of BPD, revealing substantial uptake in atherosclerotic vessels. These results suggest that BPD-MA could be a promising candidate for photodynamic therapy in the treatment of atherosclerosis [178].

A clinical study aimed to assess the safety and efficacy of local delivery of a photosensitizer followed by PDT for reducing in-stent restenosis. The study utilized Porfimer sodium delivered via a local delivery catheter, followed by pulse laser irradiation. The 18-month clinical follow-up revealed no adverse events, indicating that PDT with local delivery of Porfimer sodium is safe and may be a feasible technique for preventing in-stent restenosis [179].

In conclusion, the collective body of research presented here highlights a diverse array of strategies and promising outcomes in the utilization of photodynamic therapy for the treatment of atherosclerosis. The emphasis on targeted photosensitizers, nanoparticle formulations, and optimal treatment conditions underscores the evolving landscape of therapeutic interventions for atherosclerosis. While the results are indeed encouraging, further research and clinical trials are imperative to validate and translate these findings into effective and safe clinical interventions for patients with atherosclerosis. The interdisciplinary nature of these investigations, integrating aspects of nanotechnology, chemistry, and clinical medicine, holds significant promise for the future development of innovative therapeutic approaches in the ongoing battle against atherosclerosis.

## 4. Comparative Analysis of Costs and Side Effects: Photodynamic Therapy vs. Statin Therapy in Atherosclerosis

Atherosclerosis, a chronic and complex vascular disease, necessitates a nuanced approach to treatment. Photodynamic therapy and statin therapy represent two distinct modalities with different mechanisms of action. This comparative analysis aims to explore and contrast the costs and side effects associated with these therapeutic options in the management of atherosclerosis. In comparing the costs and side effects of PDT and statin therapy in atherosclerosis, several factors come into play. PDT offers a targeted, localized approach with potential economic implications and specific skin-related side effects. On the other hand, statin therapy, with its well-established safety profile, presents a cost-effective and systemic strategy for managing atherosclerosis. The decision between these modalities should be guided by a comprehensive assessment of individual patient needs, disease characteristics, and overall therapeutic goals. Further research and clinical experience will continue to refine our understanding of the comparative aspects of these treatment options.Table 2 shows difference between PDT and statin therapy. 

## 5. Conclusions

The application of PDT in the context of atherosclerosis holds significant promise as a novel and potentially effective treatment approach. Atherosclerosis, a complex and chronic inflammatory condition, poses a substantial global health burden, necessitating innovative therapeutic strategies. PDT, leveraging the synergistic effects of light, photosensitizing agents, and molecular oxygen, offers a targeted and minimally invasive solution. The ability of PDT to selectively target atherosclerotic plaques, reduce inflammation, and induce localized cell death highlights its potential to mitigate the progression of atherosclerosis. The technique’s non-invasiveness and specificity contribute to its appeal, minimizing collateral damage to surrounding healthy tissues. Additionally, PDT’s ability to modulate the inflammatory response and promote plaque stabilization underscores its potential in preventing plaque rupture, a critical event in atherosclerosis-associated complications like myocardial infarction and stroke. However, while the preclinical and early clinical studies are promising, further research is essential to establish the long-term safety, efficacy, and optimal treatment parameters of PDT for atherosclerosis. Overcoming challenges such as the penetration depth of light in vascular tissues and refining the delivery systems will be crucial for translating PDT into routine clinical practice. The application of photodynamic therapy in atherosclerosis represents an exciting frontier in cardiovascular medicine. Continued research and clinical trials are imperative to validate its efficacy, optimize protocols, and address practical challenges. If successful, PDT could emerge as a valuable addition to the therapeutic arsenal against atherosclerosis, offering a targeted and minimally invasive approach to combat this prevalent cardiovascular disease.

## Figures and Tables

**Table 1:** Common nanoparticles in atherosclerosis.

Examples of Nanoparticles in Atherosclerosis with a Brief Description of Use	
**High-Density Lipoprotein (HDL) Mimicking Nanoparticles**	Nanoparticles are designed to mimic the structure and function of HDL, often loaded with anti-inflammatory or antioxidant agents. They can target atherosclerotic plaques to deliver therapeutic payloads [137].
**Superparamagnetic Iron Oxide Nanoparticles (SPIONs)**	Used for imaging purposes in magnetic resonance imaging (MRI). SPIONs can be functionalized with targeting ligands for specific binding to atherosclerotic plaques, enabling non-invasive imaging [138].
**Gold Nanorods**	Utilized for both imaging and therapy. Gold nanorods can absorb near-infrared light, enabling photothermal therapy to target and treat atherosclerotic plaques [139].
**PLGA (Poly(lactic-co-glycolic acid)) Nanoparticles**	Biodegradable polymeric nanoparticles that can encapsulate drugs for sustained release. PLGA nanoparticles have been investigated for the targeted delivery of anti-inflammatory drugs to atherosclerotic lesions [140].
**Perfluorocarbon Nanobubbles**	Used as contrast agents for imaging, nanobubbles can be designed to target atherosclerotic plaques. They have been explored for ultrasound imaging to detect and monitor plaque progression [141].
**Mesoporous Silica Nanoparticles**	Designed to carry therapeutic agents and deliver them to specific locations. Mesoporous silica nanoparticles can be functionalized for targeted drug delivery to atherosclerotic lesions [142].
**Nanoparticle-Mediated Gene Therapy**	Nanoparticles can be used to deliver therapeutic genes to cells within atherosclerotic plaques. This approach aims to modulate the expression of specific genes to mitigate inflammation or promote plaque stabilization [143].
**Targeting Peptide-Modified Nanoparticles**	Nanoparticles functionalized with peptides that have an affinity for molecules overexpressed in atherosclerotic plaques. This enhances the nanoparticles’ ability to target and accumulate at specific sites [144].

**Table 2:** Costs and side effects: photodynamic therapy vs. statin therapy.

	PDT	Statin Therapy
**Costs**	Photodynamic therapy involves the administration of photosensitizers and the use of specialized light sources for activation. The costs associated with PDT can be relatively high due to the need for specific equipment, skilled personnel, and the development or acquisition of photosensitizing agents. Additionally, repeated sessions may be required for optimal efficacy, contributing to the overall economic burden.	Statin therapy is a well-established and widely prescribed approach for managing atherosclerosis. The costs associated with statins are generally lower compared to PDT. Statins are available in generic forms, contributing to cost-effectiveness. However, the overall economic impact may vary depending on the specific statin prescribed, patient adherence, and the need for additional cardiovascular medications.
**Side effects**	While PDT is generally considered a localized and targeted therapy, side effects can occur. Common side effects include photosensitivity reactions, skin irritation, and temporary discoloration of the treated area. The specificity of PDT for atherosclerotic plaques minimizes systemic side effects, but the potential for skin reactions remains a consideration.	Statins are generally well-tolerated, with a favorable safety profile. Common side effects include muscle pain or weakness, gastrointestinal disturbances, and liver enzyme abnormalities. Serious side effects, such as rhabdomyolysis, are rare but can occur. Regular monitoring of liver function and muscle health is recommended during statin therapy to mitigate potential adverse effects.
**Effectiveness**	PDT may be more suitable for localized and specific interventions, aimed at the ablation of localized plaques.	Statins provide a broader systemic approach suitable for chronic management, primarily addressing systemic factors, including cholesterol levels and inflammation.
**Long-term considerations**	The long-term safety and efficacy of PDT, especially concerning repeated treatments over extended periods, require further investigation.	Statin therapy has an extensive track record of long-term safety and efficacy, supported by numerous clinical trials and real-world evidence.

## Data Availability

All data has been included.
